# Genome-wide identification and expression analysis of serine proteases and homologs in the silkworm *Bombyx mori*

**DOI:** 10.1186/1471-2164-11-405

**Published:** 2010-06-24

**Authors:** Ping Zhao, Gen-Hong Wang, Zhao-Ming Dong, Jun Duan, Ping-Zhen Xu, Ting-Cai Cheng, Zhong-Huai Xiang, Qing-You Xia

**Affiliations:** 1 The Key Sericultural Laboratory of Agricultural Ministry, Southwest University, Chongqing 400715, PR China; 2 The Institute of Agricultural and Life Sciences, Chongqing University, Chongqing 400044, PR China

## Abstract

**Background:**

Serine proteases (SPs) and serine proteases homologs (SPHs) are a large group of proteolytic enzymes, with important roles in a variety of physiological processes, such as cell signalling, defense and development. Genome-wide identification and expression analysis of serine proteases and their homologs in the silkworm might provide valuable information about their biological functions.

**Results:**

In this study, 51 SP genes and 92 SPH genes were systematically identified in the genome of the silkworm *Bombyx mori*. Phylogenetic analysis indicated that six gene families have been amplified species-specifically in the silkworm, and the members of them showed chromosomal distribution of tandem repeats. Microarray analysis suggests that many silkworm-specific genes, such as members of SP_fam12, 13, 14 and 15, show expression patterns that are specific to tissues or developmental stages. The roles of SPs and SPHs in resisting pathogens were investigated in silkworms when they were infected by *Escherichia coli*, *Bacillus bombysepticus*, *Batrytis bassiana *and *B. mori nucleopolyhedrovirus*, respectively. Microarray experiment and real-time quantitative RT-PCR showed that 18 SP or SPH genes were significantly up-regulated after pathogen induction, suggesting that SP and SPH genes might participate in pathogenic microorganism resistance in *B. mori*.

**Conclusion:**

Silkworm SP and SPH genes were identified. Comparative genomics showed that SP and SPH genes belong to a large family, whose members are generated mainly by tandem repeat evolution. We found that silkworm has species-specific SP and SPH genes. Phylogenetic and microarray analyses provide an overview of the silkworm SP and SPHs, and facilitate future functional studies on these enzymes.

## Background

Serine proteases (SPs) in the S1 family are involved in physiological processes including digestion, development and defense [1-3]. X-ray crystallography studies show that the active center of bovine chymotrypsin is Ser195, His57, and Asp102 [4,5]. A substrate binding cleft near the active site is the predominant factor in determining SP substrate specificity. SPs are divided into several types, including the trypsin, chymotrypsin and elastase, based on the target scissile bond. SPs are usually produced as zymogens. SP zymogens are sequentially activated in a cascade pathway, with a classic example being the blood clotting cascade and complement system in mammalian plasma. Serine protease homologs (SPHs) are similar to SPs in amino acid sequence, but they have no protease activity because of losing one or more of the catalytic residue [3,4]. In insects, SPHs participate in the innate immune response [6,7].

Genome-wide analysis has been performed for SPs and SPHs in *Drosophila melanogaster *[5]. Immunity-related SPs and SPHs have been investigated in *Anopheles gambiae *and *Apis mellifera *[3,8]. However, little is known about these proteins in the silkworm, even though its SPs and SPHs are involved in biological processes that include digestion, development and immune response [9-12]. Tanaka et al. identified potential immunity-related silkworm SPs and SPHs [13], but their roles require further investigation. Changes in mRNA level during immune response are considered evidence that a gene is involved in an immune process. In this paper, we report the genome-wide classification, distribution and expression profiling of *B.mori *SPs and SPHs based on the silkworm genome and microarray data [14,15]. The role of SP and SPH genes in immunity has aslo been investigated. The results are expected to stimulate in-depth analysis of silkworm SPs and SPHs.

## Results and discussion

### Identification and chromosome distribution of silkworm SP and SPH genes

SP and SPH genes from *Manduca sexta *and other insects were used to search the silkworm genomic sequence, which revealed 143 serine protease genes. Analysis of active sites showed that 51 serine protease genes have the three active site residues intact (SP). The rest 92 serine protease genes had mutations in the catalytic residues (SPH), so they may have lost catalytic function (Additional file 1 and 2). Compared to 147 SP genes and 57 SPH genes in *Drosophila*, silkworm has 96 fewer SP genes and 35 more SPH genes. The genes were named BmSP(H)1-BmSP(H)143 (Additional file 1), 20 of which had previously been submitted to GenBank, including ovarian serine protease (GenBank Accession No. AAL62027), cocoonase (GenBank Accession No. ABR14241), vitellin-degrading protease precursor (GenBank Accession No. BAA03758), and alkaliphilic serine protease P-Iic (GenBank Accession No. AAB26023) (Additional file 3), which function in digestion, ovary and embryo development, and formation of fibroin. The remaining 123 genes were not reported previously. The genes reported here represent the first catalog of silkworm SP and SPH genes. Approximately 80 (55.9%) were found to be expressed using expressed sequence tag (EST) data according to stringent criteria (see Materials and Methods). The molecular weights and isoelectric points of silkworm SPs and SPHs were widely distributed. Predicted molecular weights were 6.3 kDa-203.6 kDa, and predicted isoelectric points were 3.5-11.3.

In arthropods, clip-domain serine proteases and homologs (clip-SPs and clip-SPHs) mediate innate immunity and embryonic development [13,14], and are involved in signal-amplifying reactions [2]. Previously, these proteases had been found only in invertebrates. We identified 7 clip-SPs and 11 clip-SPHs in the silkworm [13] (Additional file 4), which are consisted of a chymotrypsin-like SP domain, and one or more clip domain(s) at the N-terminus. The SP domain amplies signals by cleaving and activating a downstream SP [16]. Activation of the Toll pathway and melanization mediated by SP cascades are the major defense mechanisms in insects [17,18].

**Figure 1:**
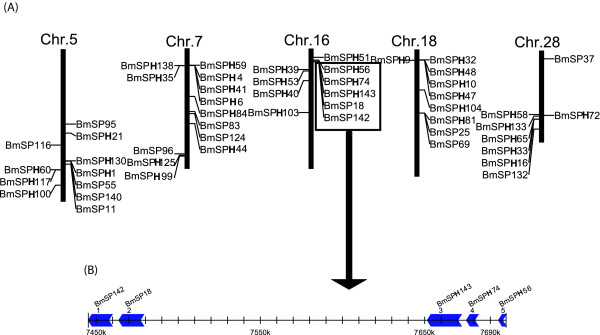
**Distribution of SP and SPH genes on silkworm chromosomes **. (A) The five Chromosomes that have the most number of SP and SPH genes. Many SP and SPH genes are in tandem clusters. (B) An example of SP and SPH genes clustered on chromosome 16. These five genes are distributed in a cluster within 250 kb.The arrowhead indicates the transcriptional orientation of genes.

The updated silkworm genome has 128 SP and SPH genes (89.5%) on 28 chromosomes, with an uneven distribution. For example, chromosome 1 and 19 have no SP and SPH genes, while both chromosomes 17 and 22 have one. However, chromosome 5 has 11 genes, chromosome 7 has 13, and chromosome 16 has 10 (Figure 1). Additional analysis showed that SP and SPH genes exhibit a tandem repeat distribution on the chromosomes. For example, there are 10 genes in nscaf3058 of chromosome 16, and *BmSP18, BmSPH56, BmSPH74, BmSP142 *and *BmSPH143 *showed tandem repeat distribution on this chromosome (Figure 1). Of these, *BmSPH74 *and *BmSP143 *were closely linked. The distance between the two genes is only 3 kb, and their sequence identities were up to 96%. Therefore, these genes probably arose by gene duplication.

### Species-specific SP and SPH genes in the silkworm

The TRIBE-MCL clustering algorithm was used to group the SP and SPH genes into families. *Caenorhabditis elegans*, *Homo sapiens, Drosophila melanogaster, Apis mellifera and Anopheles gambiae *were included to find members of evolutionarily conserved families, which were defined as containing silkworm genes, and at least one gene in another species. The results uncovered 99 SP and SPH silkworm genes, belonging to 17 gene families, 11 of which were evolutionally conserved (Table 1). SP_fam1 contains the most number of SP and SPH genes. These exist in *C. elegans*, insects and humans, indicating that SP_fam1 existed before the divergence of these species. SP_fam1 has two members in *C. elegans*, and shows great expansion in insects and mammals. *B. mori *and *A. mellifera *contain 24 and 28 members of SP_fam1, respectively. However, the Diptera insects *D. melanogaster *and *A. gambiae*, have 65 and 49 SP_fam1 members, respectively. Interestingly, the other 10 evolutionarily conserved SP and SPH gene families are insect-specific.

**Table 1:** The gene number of SP and SPH gene families in different species

Family	*C. elegan*	*B. mori*	*D. melanogaster*	*A. gambiae*	*A. mellifera*	Human
SP_fam1	2	24	65	49	28	61
SP_fam2	-	9	12	37	4	-
SP_fam3	-	6	18	22	2	-
SP_fam4	-	5	17	19	4	-
SP_fam5	-	4	2	29	0	-
SP_fam6	-	2	9	9	1	-
SP_fam7	-	1	3	4	2	-
SP_fam8	-	5	-	1	0	-
SP_fam9	-	2	1	1	1	-
SP_fam10	-	1	1	1	1	-
SP_fam11	-	1	1	1	1	-
SP_fam12	-	15	-	-	-	-
SP_fam13	-	7	-	-	-	-
SP_fam14	-	6	-	-	-	-
SP_fam15	-	5	-	-	-	-
SP_fam16	-	3	-	-	-	-
SP_fam17	-	3	-	-	-	-

**Figure 2:**
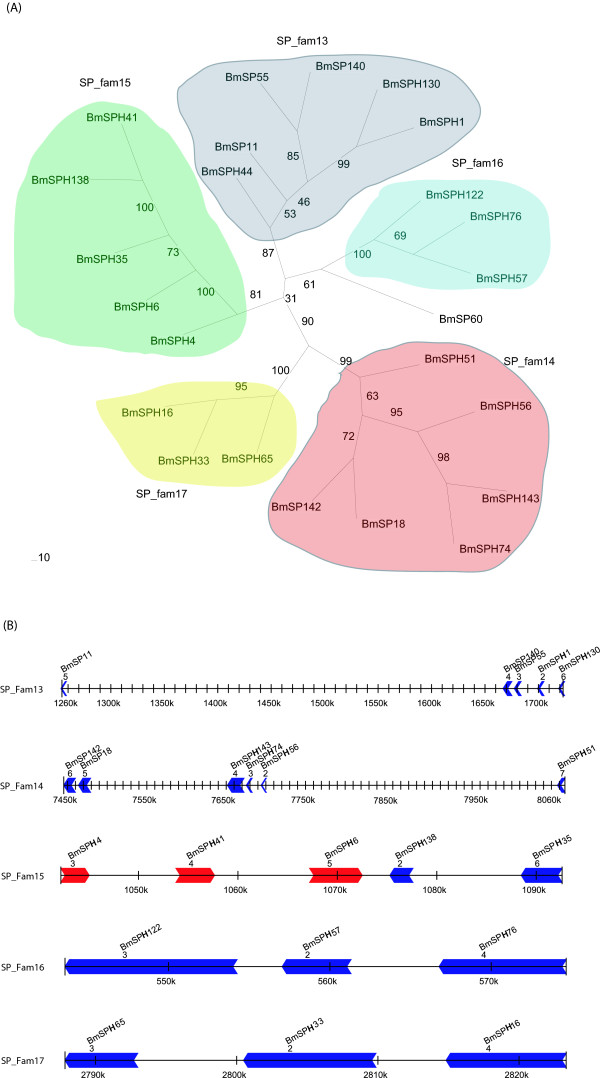
**Phylogenetic analysis of silkworm-specific SP and SPH genes **. (A) Neighbor-joining tree of silkworm-specific SP and SPH genes. Genes in SP_fam13, SP_fam14, SP_fam15, SP_fam16, SP_fam17 are in gray, red, green, blue and yellow, respectively. (B) Schematic picture of the distribution on chromosome for genes of SP_fam13, SP_fam14, SP_fam15, SP_fam16 and SP_fam17. The arrowhead indicates the transcriptional orientation of genes.

The silkworm genome contains 39 genes in 6 silkworm-specific SP and SPH gene families (Table 1 and Additional file 5). If the 44 singleton SP genes are considered, 83 species-specific SP and SPH genes exist, accounting for 56.8% of the silkworm SP and SPH genes. This indicated that the genes that came from the same species-specific SP and SPH gene families were the result of tandem repeats (Figure 2). For example, SP_fam13 has 7 members, and 5 occur as tandem repeats on chromosome 10. Internal members of the same family showed tandem repeat distribution in SP_fam14 (chr.16), SP_fam15 (chr.7), SP_fam16 (chr.26) and SP_fam17 (chr.28). Thus, tandem repeat distribution has an important effect on the expansion of species-specific SP and SPH genes in silkworm. In fact, silkworm genome analysis shows that many silkworm-specific gene families have the typical features of a chromosomal tandem repeat distribution [2,19,20]. In addition, gene sequences in the same cluster show high identity. For example, the chromosomal location of *BmSPH74 *and *BmSPH143 *is a typical tandem repeat, with the genes closely linked on the chromosome, only 3 kb apart, and sequence identity of up to 96%. This indicated that one of the genes may have emerged from the duplication of the other, and the duplication event happened recently. Therefore, these specific genes may come from a specific gene duplication family. Genes that originated by duplication might have adapted during silkworm evolution and may cut substrates specific to silkworm. However, this hypothesis need to be valitated by further experiments.

### Expression profile of silkworm SP and SPH genes

**Figure 3:**
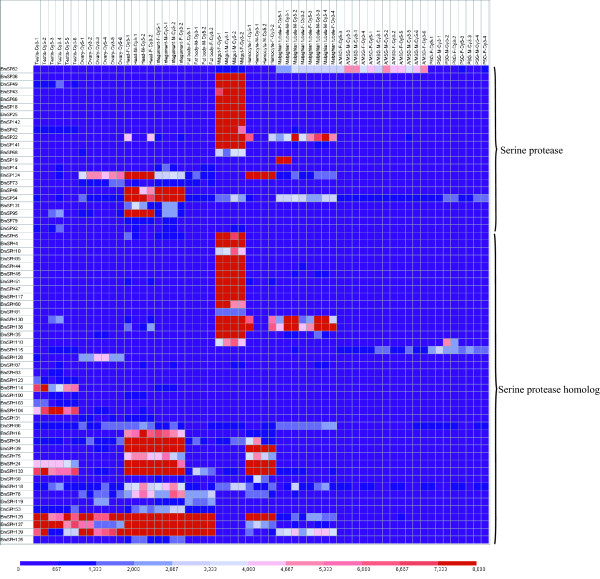
**Tissue expression profile of silkworm SP and SPH genes **. Gene expression levels are represented by red (higher expression) and blue (lower expression) boxes. The columns represents ten different tissue or organ samples: testis, ovary, head, integument, fat body, midgut, hemocyte, malpighian tubule, A/MSG(anterior/median silk gland) and PSG(posterior silk gland). The column name is composed as "sample name-labeling dye- experiment order".

**Figure 4:**
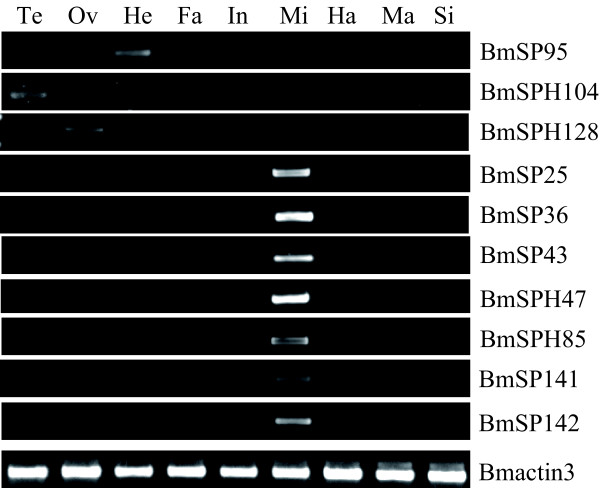
**Tissue expression patterns of some silkworm SP and SPH genes showing tissue specificity on day 3 of the fifth instar. **Semi-quantitative RT-PCR amplification of total RNA with BmSP(H)-specific oligonucleotides. Te, testis; Ov, ovary; He, head; Fa, fat body; In, integument; Mi, midgut; Ha, haemocyte; Ma, Malpighian tubules; Si, silk gland. The silkworm cytoplasmic actin A3 gene (Bmactin3; GenBank accession no. U49854) was used as internal control, and denoted by *Bmactin3*.

Microarray analysis showed that 64 SP and SPH genes have transcriptional activity in at least one tissue or organ according to a stringent criterion for definition of expressed genes [15]. Midgut had the highest number (38) of expressed genes among the 10 tissues or organs, while the number of expressed genes in testis, ovary, head, integument, fat body, hemocytes, Malpighian tubule, A/MSG and PSG was 26, 24, 32, 30, 15, 22, 20, 7 and 6, respectively (Figure 3). Further analysis reveals that most of genes expressed highly and exclusively in the midgut belong to silkworm-specific families, such as SP_fam12, SP_fam13, SP_fam14 and SP_fam15. These gene families showed unique midgut expression feature that are likely to reflect the special food habit of silkworm, and the process of accumulating abundant amino acids for producing silk. It may also be associated with resistance with pathogenic microorganisms. Some genes had unique expression features in other tissues on the third day of the silkworm fifth instar larva according to microarray analysis, for example, exclusive expression was seen for *BmSP95 *in the head, *BmSPH104 *in the testis, *and BmSPH128 *in the ovaries. Semi-quantitative RT-PCR was also performed to confirm tissue expression patterns of some representative SP and SPH genes for day-3 fifth instar larvae. The results were the same as those for the microarray data (Figure 4).

**Figure 5:**
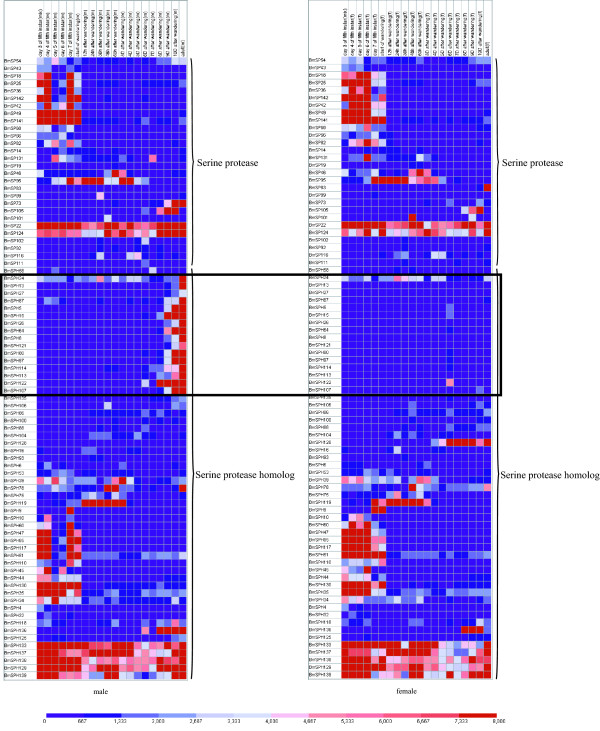
**Expression profile of SP and SPH genes during different developmental stages. **Gene expression levels are represented by red (higher) and blue (lower) boxes. Genes that express only in male silkworm during metamorphosis are marked by a rectangle. The columns represents twenty different sample time points: day 3, 4, 5, 6, 7 of the fifth instar, start of wandering, 13 different times after wandering: 12 hour, 24 hours, 36 hours, 48 hours, 60 hours, 3 days, 4 days, 5 days, 6 days, 7 days, 8 days, 9 days, 10 days and adult.Word "mix" in the column name represents that the sample came from both gender; word "m" in the column name represents that the sample came from male silkworm; word "f" in the column name represents that the sample came from female silkworm.

More than 20 genes are expressed exclusively at the fifth instar larval stage, and about 7 genes are expressed highly over the entire metamorphosis process (Figure 5). The majority of SP and SPH genes express in both male and female silkworms. However, a group of SPH genes show differences in expression between males and females during the metamorphosis in which a pupa turns into an adult. These genes express highly and exclusively in males, but are minimally expressed in females during metamorphosis (Figure 5).

We analyzed the expression pattern of repeated genes using information on gene location and expression data from tissue microarray experiments, and found that repeated SP and SPH gene groups in silkworm do not have identical expression patterns. For example, the repeated gene group on chr.16 includes several genes, but only *BmSP18 *and *BmSP142 *have a similar expression pattern. Of 23 SP and SPH genes expressed exclusively in the midgut, only *BmSP18 *and *BmSP142 *are tandem repeat genes, with too much distance between the other genes (Figure 2), although some were located on the same chromosome, such as *BmSPH35, BmSPH4, BmSPH44 *and *BmSPH6 *on chr.7; *BmSPH10, BmSP25, BmSPH47 *and *BmSPH81 *on chr.18; *BmS142, BmS18 *and *BmSPH51 *on chr.16; and *BmSP141 *and *BmSP42 *on chr.12. These results were confirmed by developmental expression patterns (data not shown)

**Figure 6:**
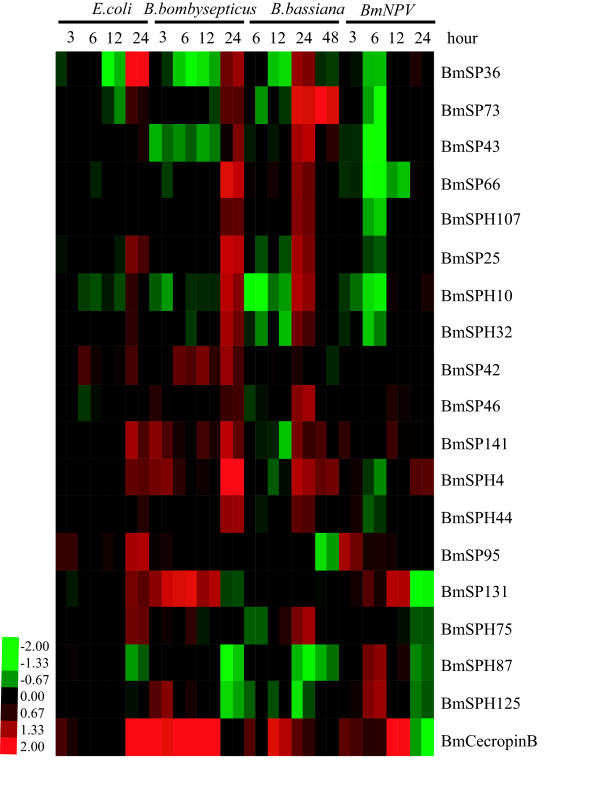
**Hierarchical cluster analysis of SP and SPH genes after microorganism infection. **Red represents that gene expression is up-regulated; black represents that gene expression is not changed; green represents that gene expression is download-regulated; gray represents data missing. Hierarchical clustering was performed using Cluster version 3.0 and visualized by TreeView.

To investigate whether silkworm SP genes are involved in the immune response, SP and SPH expression changes were analyzed after insects infected by four different microorganisms. We found that expression of 65 SP and SPH genes could be detected. Among these, 18 were up-regulated more than 2.0 times in the infected group compared to the control group (Figure 6 and Additional file 6). Most were up-regulated most strongly during the late stage, 24 hours after infection. For example, *BmSP36 *expression was up to 4 times higher than the control at 24 hours after *E. coli *infection, and twice as high as the 24-h point for *Bacillus bombysepticus *or *Batrytis bassiana *infection. Expression of *BmSPH4 *exceeded 1.5 times the control after infection with any of the four microorganisms. A few genes showed expression regulation that was different, depending on the infecting microorganism. For example, *BmSPH125 *was up-regulated after infection with *E.coli, B.bombyspticus *or *B. mori nucleopolyhedrovirus *(BmNPV), but did not change significantly after infection with *B.bassiana*. However, when we compared the magnitude in induction of gene expression between antibacterial proteins *BmcecropinB *and silkworm SP or SPH genes, we found that *BmcecropinB *was remarkably upregulated on infection by these four pathogens at all time points except for BmNPV infection at 24 h (Figure 6). The induced expression pattern was consistent with SP or SPH genes at most of the time points. The data suggest that SP or SPH genes might play important roles in the innate immune system of *B. mori*. The gene expression results from microarray experiments were validated by real-time quantitative RT-PCR (Additional file 7). The results are consistent with the microarray experiments. Only 3 of the 18 up-regulated genes were in GenBank, specifically BmSPH125 (NP_001036891), BmSP131 (NP_001040537) and BmSP141 (NP_001040178) (Additional file 3).Of 18 up-regulated SPs and SPHs, BmSP95 has two clip domains, while BmSPH125 has one clip domain (Additional file 4). Although the function of clip domains is not yet clear, enzymes that contain this structure are of interest, because they may regulate serine protease activity and mediate innate immunity [5].

SPs involved in cascade regulation of the insect immune response were found to be highly conserved. Broehan found that chymotrypsin-like protease cascade systems that contain CTLP1 control chitin synthase activity in the tobacco hornworm, *Manduca sexta *[21]. The chitin portion of the peritrophic matrix in the midgut plays an important role in resistance to pathogenic bacteria and toxins. BmSPH10 has 70% identity to CLIP 1 (Additional file 6), and like CLIP 1, BmSPH10 was also expressed in the midgut, so it may have a similar function. The melanism reaction in insects is mediated by an SP signalling cascade that is highly conserved. In the *M. sexta *SP signalling cascade, proHP14 is a pattern recognition protein that binds to bacteria and autoactivates and triggers the prophenoloxidase activation system in the hemolymph, which contains a series of proteinases such as HP1, 6 8, 17 and 21. Finally prophenoloxidase-activating proteinase (PAPs) are activated, leading to the melanism reaction [22-24]. Cytotoxic molecules are produced in this process, including quinones and reactive oxygen intermediates, that may kill the invading microorganisms that are trapped by the melanin [25]. BmSP14, BmSPH125 and BmSPH78 in the silkworm are highly similar to HP14, HP17 and PAP, respectively, in the tobacco hornworm (Additional file 6). BmSP14, BmSPH125 and BmSPH78 were markedly up-regulated after induction, suggesting that they may have similar functions to their tobacco hornworm homologs (BmSP14 and BmSPH78, which were up-regulated between 1.5 and 2 times, were not included in the table).

## Conclusions

From the updated *B. mori *silkworm genome, we identified SP and SPH genes and analyzed their expression patterns. Comparative genomics showed that SP and SPH genes belong to a large family whose members were generated mainly by duplication and show a lineage of tandem chromosomal repeats. In addition, we found that silkworm has species-specific SP and SPH genes, and combining the results of express profiles, we hypothesize that members of the silkworm SP and SPH families may participate in biological processes that include digestion, development and the immune response. We hope the data presented in this study facilitates functional studies on SPs and SPHs in *B. mori*.

## Methods

### Identification of silkworm SP and SPH genes in the silkworm

SP and SPH genes from *M. sexta *and other insects were downloaded from GenBank http://www.ncbi.nlm.nih.gov/Genbank/. The sequence homology BLAST alignment tool was downloaded from the ftp site of National Center for Biotechnology Information ftp://ftp.ncbi.nih.gov/blast/. SP and SPH gene sequences from other insects were used as queries in BLAST searches against the silkworm database (E-value: 10^-6^) [26]. Identified genes were validated by aligning to a non-redundant gene dataset. Identified genes were also validated by BLAST searching against an EST database with threshold E-value <10^-30^, identities >90%, and match lengths >100 bp.

### Evolutionary analysis of silkworm SP and SPH genes

SP and SPH sequences of *D. melanogaster, C. elegans *and *A. gambiae *were included in an all-against-all BLAST search using BLASTP (E-value 1e^-30^), with the results converted into a Markov Martrix [27]. The TRIBE-MCL clustering algorithm was used to detect family members [27]. Clustering operations were implemented at inflation values of 1.2. Identified SP and SPH genes were used for phylogenetic analyses. Multiple sequence alignments of protein sequences were made using ClustalX 1.83 [28], and neighbor-joining used Phylip software [29], with a bootstrap value of 1000 to reconstruct the phylogenetic tree.

### Expression of silkworm SP and SPH genes from whole-genome microarrays

In 2007, Xia et al. constructed a genome-wide oligonucleotide microarray with more than 22,000 probes [15], including 107 SP- and SPH-specific oligonucleotide probes. We analyzed tissue expression of SP and SPH genes at the third day of the fifth instar larvae of *Dazao*. In brief, insects were reared at a stable temperature of 25°C until the third day of the fifth instar. Then testis, ovary, head, integument, fat body, midgut, hemocytes, Malpighian tubules, A/MSG (anterior/median silk gland) and PSG (posterior silk gland) from this developmental point were hand-dissected on ice. To determine the developmental expression patterns, individuals at 20 different time points from day 3 of the fifth instar to the moth stage were collected for both genders. Gene expression levels were visualized using GeneCluster 2.0 [30]. The detailed experimental procedures for microarray and data analyses were as previously reported [15].

For insects used for microorganism induction, newly hatched Dazao larvae were reared to the third day of the fifth instar with an artificial diet, in constant-temperature incubators. The gram-negative *E. coli*, gram-positive *B. bombysepticus*, *B. bassiana *and BmNPV were used for feeding induction, and Dazao fed with physiological saline were used as negative control. Whole body of individual samples induced with *B. bassiana *were collected after 6, 12, 24 and 48 hours, and whole body of individual samples from induction with the other microorganisms were collected after 3, 6, 12 and 24 hours. Microarray procedures and data analysis were as described [15]. Genes were considered to be up-regulated if the change in gene expression was greater than two fold on at least one occasion over the four time points in any of the four microorganism induction experiments [31-35]. Hierarchical clustering of gene expression patterns was performed using the Cluster (version 3.0) and visualized by TreeView programs [36].

### Tissue expression analysis of silkworm SP and SPH genes by semi-quantitative RT-PCR

Total RNA was isolated from nine tissues (testis, ovary, head, fat body, integument, midgut, hemocytes, Malpighian tubules and silk gland) from day 3 fifth instar larvae using TRIzol reagent (Invitrogen, USA). Contaminating genomic DNA was digested using RNase-free DNase I (Promega) for 30 min at 37°C. RNA samples were diluted with RNase-free water and stored at -80°C. The concentration of total RNA was estimated by measuring the absorbance at 260 nm. Total RNA (10 μg) was reverse-transcribed into cDNA using M-MLV reverse transcriptase (Invitrogen, USA) at 42°C. All cDNA samples were normalized using *B. mori *actin A3 as an internal control (forward primer: 5'-AAC ACC CCG TCC TGC TCA CTG-3'; reverse primer: 5'-GGG CGA GAC GTG TGA TTT CCT-3'). All SP and SPH primers for semi-quantitative RT-PCR detection are listed in Table Additional file 8. PCR amplification was performed in a total reaction volume of 25 μL containing normalized cDNA, 10 pmol of each primer, 2 mM MgCl_2_, 0.25 mM dNTP, 1× buffer and 2.5 U of Taq DNA polymerase. Semi-quantitative RT-PCR reactions were performed in a volume of 25 μL using the following program: initial incubation at 94°C for 4 min, followed by 24-28 cycles (BmSP95, BmSPH104, BmSPH128, BmSP36, BmSPH47, BmSPH85, BmSP142 for 24 cycles; BmSP43 and BmSP141 for 26 cycles; BmSP25 for 28 cycles) of 40 s at 94°C, 40 s of annealing (temperatures listed in Additional file 8), 45 s of extension (72°C), and a final extension at 72°C for 10 min. Aliquots of 4 μL of the PCR products were separated on 2.0% agarose gels and stained with EB.

### Induced expression analysis of silkworm SP and SPH genes by real-time quantitative RT-PCR

Quantitative RT-PCR was performed using an ABI PRISM 7000 sequence detection system (Applied Biosystems). The 15 μl mixture included 1.5 μl of cDNA, 0.5 mM of each primer and 1× SYBR Premix Ex Taq (TaKaRa) in each well of a 96-well plate. PCR was carreied out with initial denaturation at 94°C for 10 s, followed by 40 cycles at 95°C for 5 s and 60°C for 31 s. The primer sequences for all genes are listed in Additional file 9. Relative gene expression data were normalized against Ct values for the housekeeping sw22934 gene in silkworm and the fold change (2^-ΔΔCt^) was determined by comparison with average expression levels for control samples, with the index dened as 1.0. Four larvae were used for each treatment. Each expression assay was repeated three times. Student's t-test was used to evaluate statistical signicance (P < 0.01).

## Authors' contributions

PZ conceived and designed the study and wrote the manuscript. GHW performed microarray experiments and generated array data. ZMD and JD identified the SPs and SPHs and analyzed the sequences. PZX and TCC performed microorganism induction experiments. QYX participated in designing the study, analyzed the results and revised the manuscript. ZHX supervised the study. All authors read and approved the final manuscript.

## Supplementary Material

Additional file 1 **The SPs and SPHs predicted in the silkworm **. a. The scaffold name of silkworm genome that can be found in the SilkDB http://www.silkdb.org; b. The start position of the SPs and SPHs gene on the silkworm genome; c. The end position of the SPs and SPHs gene on the silkworm genome; d. The strand of the SPs and SPHs gene on the silkworm genome; e. The position of the SPs and SPHs gene on the silkworm chromosomes; f. Whether the SPs and SPHs genes are supported by the EST evidence. Click here for file

Additional file 2 **The serine protease genes that contain intact active site **. The active site of Ser, His, and Asp play important role in the catalytic processes for SP genes. By analyzing the gene sequences, we found that 51 serine protease genes have the three active site residues intact (SP), and the rest 92 serine protease genes had mutations in the catalytic residues (SPH), so they may have lost catalytic function. Click here for file

Additional file 3 **The SPs and SPHs in the silkworm that have been reported **. The GenBank accession numbers and description of the reported SPs and SPHs in the silkworm are listed. Click here for file

Additional file 4 **The clip domain in the silkworm, *Bombyx mori***. The conserved six cysteine residues for each CLIP are marked by color. We identified 7 clip-SPs and 11 clip-SPHs in the silkworm, which are consisted of a chymotrypsin-like SP domain, and one or more clip domain(s) at the N-terminus. Click here for file

Additional file 5 **Phylogenetic analysis of silkworm-specific SP and SPH genes of silkworm and *Drosophila***. Most of **t **he SP and SPH genes were included in the analysis. Except for SP_fam1, we choose three representative genes to analyze. Multiple alignments of protein sequences were made by ClustalW. Then neighbor-joining phylogenetic trees were reconstructed by Phylip. The parameters were chosen as follows: the evolutionary distance was poisson-corrected, gaps were completely deleted, and 100 iterations were used for calculating bootstrap values. Click here for file

Additional file 6 **The up-regulated SP and SPHs after silkworm infected by microorganisms **. Genes whose expression were up-regulated than two fold on at least one occasion over the four time points in any of the four microorganism induction experiments were listed. The best NCBI BLAST Results of the up-regulated SP and SPHs were also shown in the table. Click here for file

Additional file 7 **The induced expression analysis of silkworm SP and SPH genes by quantitative real-time RT-PCR **. We chose the time points of infecting 6 h and 24 h to do the expression analysis. The expression of SP or SPH in the control sample was set to 1. The abbreviations are used, the *E. coli *infected sample (Ec), the *B. bombyseptieus *infected sample (Bs), the *B. bassiana *infected sample (Bb) and the *B. mori nucleopolyhedrovirus *infected sample (NPV). Each expressive assay was replicated by three times. The Student's t-test was used to evaluate statistical signicance (P < 0. 01). Click here for file

Additional file 8 **Primers used in semi-quantitative RT-PCR study **. Primer sequences, melting temperature, and amplicon size were listed. Click here for file

Additional file 9 **Primers used in qRT-PCR study **. Primer sequences, melting temperature and amplicon size were listed. Click here for file
