# Supplementary material for: Genome-wide identification and expression analysis of serine proteases and homologs in the silkworm Bombyx mori
Source: BMC Genomics. 2010 Jun 24;11:405. doi: 10.1186/1471-2164-11-405 (PMC2996933; doi:10.1186/1471-2164-11-405)
Supplement: Additional file 1 — The SPs and SPHs predicted in the silkworm . a. The scaffold name of silkworm genome that can be found in the SilkDB http://www.silkdb.org; b. The start position of the SPs and SPHs gene on the silkworm genome; c. The end position of the SPs and SPHs gene on the silkworm genome; d. The strand of the SPs and SPHs gene on the silkworm genome; e. The position of the SPs and SPHs gene on the silkworm chromosomes; f. Whether the SPs and SPHs genes are supported by the EST evidence. [file 1471-2164-11-405-S1.DOC]

| Name | Molecular weight | Isoelectric point | Scaffold # | Starting point | End point | Chain | Chr.# | EST |
| --- | --- | --- | --- | --- | --- | --- | --- | --- |
| BmSPH1 | 27991.48 | 9.9394 | nscaf2674 | 1700964 | 1703335 | - | 5 | Yes |
| BmSP2 | 31398.29 | 9.9161 | scaffold644 | 923 | 2605 | - | / | Yes |
| BmSPH3 | 24901.33 | 4.6136 | nscaf2823 | 4221690 | 4226763 | + | 25 | No |
| BmSPH4 | 21687.38 | 4.7181 | nscaf2983 | 1042201 | 1045047 | + | 7 | No |
| BmSPH5 | 35295.3 | 8.1501 | nscaf2795 | 2738748 | 2745669 | + | 20 | No |
| BmSPH6 | 27568.19 | 4.5607 | nscaf2983 | 1067217 | 1072501 | + | 7 | No |
| BmSP7 | 25670.88 | 9.3216 | nscaf2953 | 3401675 | 3403521 | + | 14 | Yes |
| BmSPH8 | 22079.43 | 9.0973 | nscaf3138 | 25241 | 28740 | + | 27 | No |
| BmSPH9 | 32120.46 | 4.5907 | nscaf2901 | 1296218 | 1300543 | - | 18 | No |
| BmSPH10 | 56217.69 | 6.9966 | nscaf2901 | 1255939 | 1270547 | + | 18 | Yes |
| BmSP11 | 27598.42 | 9.1044 | nscaf2674 | 1254259 | 1257447 | - | 5 | No |
| BmSP12 | 29850.73 | 4.6852 | nscaf1898 | 15651205 | 15657866 | - | 13 | Yes |
| BmSPH13 | 26785.13 | 9.3675 | nscaf2589 | 2292140 | 2293914 | + | 4 | No |
| BmSP14 | 31383.46 | 6.6563 | nscaf3038 | 162459 | 167302 | - | / | Yes |
| BmSPH15 | 28355.44 | 7.0429 | nscaf3089 | 259642 | 265958 | - | 20 | No |
| BmSPH16 | 47252.36 | 4.8782 | nscaf3097 | 2814831 | 2823335 | - | 28 | No |
| BmSPH17 | 24697.21 | 3.914 | nscaf3038 | 176486 | 184091 | - | / | No |
| BmSP18 | 27779.48 | 6.7668 | nscaf3058 | 7463822 | 7479028 | - | 16 | Yes |
| BmSP19 | 29606.38 | 10.7859 | scaffold644 | 47673 | 51571 | + | / | Yes |
| BmSP20 | 28975.11 | 8.0644 | nscaf3093 | 439152 | 444821 | + | 13 | No |
| BmSPH21 | 28691.59 | 7.2402 | nscaf2674 | 5585962 | 5590593 | - | 5 | No |
| BmSP22 | 29679.1 | 10.5005 | scaffold644 | 11381 | 13100 | + | / | Yes |
| BmSP23 | 41664.91 | 8.6959 | nscaf2964 | 1809463 | 1817972 | - | 2 | No |
| BmSPH24 | 39059.75 | 7.4522 | scaffold606 | 79314 | 88808 | + | 24 | Yes |
| BmSP25 | 30907.08 | 8.0544 | nscaf2902 | 7055765 | 7058315 | + | 18 | Yes |
| BmSPH26 | 15960.86 | 4.6548 | nscaf3090 | 231500 | 235894 | - | / | No |
| BmSPH27 | 50972.58 | 9.3225 | nscaf3089 | 277823 | 283952 | - | 20 | Yes |
| BmSPH28 | 12191.9 | 8.9531 | nscaf2828 | 725251 | 726954 | - | 8 | No |
| BmSPH29 | 19462.9 | 11.3186 | nscaf2953 | 3394603 | 3395823 | + | 14 | Yes |
| BmSPH30 | 6873.97 | 7.3459 | nscaf2993 | 8087241 | 8087432 | + | 12 | No |
| BmSPH31 | 23607.81 | 4.4231 | nscaf3044 | 485409 | 490720 | - | 21 | Yes |
| BmSPH32 | 26831.47 | 6.3842 | nscaf2901 | 1238349 | 1244666 | + | 18 | No |
| BmSPH33 | 55986.17 | 5.1414 | nscaf3097 | 2800487 | 2809863 | - | 28 | No |
| BmSPH34 | 30849.19 | 6.0841 | scaffold651 | 19656 | 30575 | - | / | Yes |
| BmSPH35 | 28671.37 | 5.2357 | nscaf2983 | 1088495 | 1092596 | - | 7 | Yes |
| BmSP36 | 31388.48 | 8.1702 | nscaf2883 | 320163 | 323297 | - | 3 | Yes |
| BmSP37 | 29554.38 | 5.1942 | nscaf3099 | 943648 | 950102 | + | 28 | No |
| BmSPH38 | 15509.48 | 4.3297 | nscaf3072 | 1123546 | 1126586 | - | 27 | No |
| BmSPH39 | 54731.98 | 7.8645 | nscaf3058 | 6381499 | 6392772 | + | 16 | Yes |
| BmSPH40 | 6326.31 | 8.0638 | nscaf3058 | 4825086 | 4826394 | + | 16 | No |
| BmSPH41 | 28236.73 | 5.7299 | nscaf2983 | 1053773 | 1057648 | + | 7 | Yes |
| BmSP42 | 28283.18 | 7.8649 | nscaf2993 | 8089340 | 8091867 | + | 12 | Yes |
| BmSP43 | 29812.87 | 9.0145 | nscaf3103 | 49739 | 53165 | + | / | Yes |
| BmSPH44 | 22115.94 | 8.1913 | nscaf2986 | 5263279 | 5266417 | + | 7 | Yes |
| BmSPH45 | 29128.2 | 6.8969 | nscaf1962 | 223924 | 228029 | + | 23 | Yes |
| BmSP46 | 77928.86 | 4.5267 | nscaf3044 | 528508 | 541587 | - | 21 | Yes |
| BmSPH47 | 29896.06 | 8.3921 | nscaf2901 | 1288304 | 1294564 | + | 18 | Yes |
| BmSPH48 | 29278.14 | 7.2912 | nscaf2901 | 1247739 | 1252312 | + | 18 | Yes |
| BmSP49 | 30083.09 | 9.3713 | scaffold644 | 83952 | 86585 | - | / | Yes |
| BmSP50 | 29721.24 | 10.6361 | scaffold644 | 31830 | 39124 | + | / | Yes |
| BmSPH51 | 39933.99 | 5.7306 | nscaf3058 | 8067640 | 8076943 | - | 16 | Yes |
| BmSPH52 | 55628.95 | 7.3862 | nscaf3089 | 285563 | 290290 | + | 20 | No |
| BmSPH53 | 37568.02 | 7.8518 | nscaf3058 | 6172394 | 6178382 | + | 16 | Yes |
| BmSP54 | 37985.73 | 6.6161 | nscaf2987 | 19544 | 29543 | + | 12 | Yes |
| BmSP55 | 27776.48 | 9.3969 | nscaf2674 | 1677402 | 1683769 | - | 5 | Yes |
| BmSPH56 | 20994.66 | 6.2898 | nscaf3058 | 7694957 | 7699597 | - | 16 | No |
| BmSPH57 | 37181.71 | 7.0027 | nscaf2330 | 557044 | 561308 | - | 26 | No |
| BmSPH58 | 8335.67 | 8.4915 | nscaf3097 | 2164688 | 2164918 | - | 28 | No |
| BmSPH59 | 7411.68 | 5.565 | nscaf2983 | 1032966 | 1034944 | + | 7 | No |
| BmSPH60 | 10182.21 | 8.6432 | nscaf2674 | 478999 | 479283 | + | 5 | Yes |
| BmSP61 | 27842.77 | 8.1779 | nscaf3026 | 4640616 | 4645091 | - | 23 | No |
| BmSPH62 | 33231.11 | 7.9392 | nscaf2888 | 9915674 | 9918315 | - | 15 | Yes |
| BmSP63 | 181318.28 | 8.0191 | nscaf2962 | 458277 | 498850 | + | 24 | Yes |
| BmSPH64 | 29565.73 | 7.1285 | nscaf2589 | 2278651 | 2282012 | + | 4 | No |
| BmSPH65 | 35507.98 | 4.7628 | nscaf3097 | 2787841 | 2792992 | - | 28 | No |
| BmSP66 | 29997.96 | 6.6552 | nscaf3045 | 530322 | 536206 | - | 9 | Yes |
| BmSP67 | 28820.82 | 10.0118 | nscaf2964 | 805255 | 818532 | + | 2 | No |
| BmSP68 | 29042.62 | 7.8538 | nscaf2953 | 3379508 | 3384575 | + | 14 | Yes |
| BmSP69 | 30994.41 | 5.6319 | nscaf2902 | 7059922 | 7064937 | + | 18 | Yes |
| BmSP70 | 28580.73 | 8.4074 | nscaf2964 | 726416 | 731477 | - | 2 | No |
| BmSP71 | 43753.86 | 7.2808 | nscaf2987 | 110499 | 115016 | + | 12 | Yes |
| BmSPH72 | 29853.94 | 5.7233 | nscaf3097 | 2276197 | 2284207 | + | 28 | Yes |
| BmSP73 | 21563.52 | 8.9152 | nscaf2589 | 3708095 | 3709549 | - | 4 | No |
| BmSPH74 | 37835.76 | 4.3213 | nscaf3058 | 7675423 | 7682683 | - | 16 | Yes |
| BmSPH75 | 26725.51 | 6.1227 | nscaf3044 | 505324 | 507830 | - | 21 | Yes |
| BmSPH76 | 36718.28 | 7.992 | nscaf2330 | 566768 | 574644 | - | 26 | No |
| BmSP77 | 50605.04 | 8.0345 | nscaf2865 | 536968 | 541695 | - | 17 | Yes |
| BmSPH78 | 41356.48 | 5.4388 | nscaf2993 | 4905389 | 4919031 | + | 12 | Yes |
| BmSP79 | 33432.48 | 4.5723 | nscaf2176 | 933608 | 937329 | + | 11 | Yes |
| BmSPH80 | 16667.9 | 8.0737 | nscaf3090 | 242278 | 243163 | - | / | No |
| BmSPH81 | 21375.26 | 5.5902 | nscaf2902 | 7050339 | 7051225 | + | 18 | No |
| BmSP82 | 32989.79 | 8.033 | nscaf2795 | 1108881 | 1115723 | + | 20 | No |
| BmSP83 | 22418.66 | 8.361 | nscaf2986 | 3571523 | 3572137 | + | 7 | No |
| BmSPH84 | 16328.21 | 10.5068 | nscaf2986 | 1473450 | 1478611 | + | 7 | Yes |
| BmSPH85 | 25171.36 | 3.5368 | nscaf2868 | 62080 | 68901 | - | 21 | Yes |
| BmSPH86 | 55292.22 | 8.6322 | nscaf3087 | 126353 | 136720 | + | / | No |
| BmSPH87 | 10627.99 | 7.2904 | nscaf3093 | 450800 | 451693 | - | 13 | No |
| BmSPH88 | 56001.17 | 6.307 | nscaf2859 | 1719737 | 1724366 | + | 10 | Yes |
| BmSP89 | 33555.61 | 5.8551 | nscaf2797 | 438172 | 439080 | - | 27 | No |
| BmSP90 | 33582.84 | 10.4614 | nscaf1681 | 1225904 | 1240884 | + | 22 | No |
| BmSPH91 | 121640.33 | 6.5081 | nscaf3031 | 513090 | 517001 | - | 11 | Yes |
| BmSP92 | 69144.59 | 6.2183 | nscaf2964 | 1569105 | 1588540 | + | 2 | No |
| BmSPH93 | 50719.34 | 6.0279 | nscaf2883 | 1156243 | 1166008 | - | 3 | No |
| BmSPH94 | 21586.26 | 5.3203 | nscaf2855 | 4840077 | 4842087 | + | 10 | Yes |
| BmSP95 | 71365.49 | 5.4651 | nscaf2674 | 6836132 | 6850048 | - | 5 | Yes |
| BmSP96 | 64707.11 | 8.4624 | nscaf2912 | 942360 | 950283 | - | 7 | No |
| BmSPH97 | 23066.33 | 9.1932 | nscaf2589 | 2295633 | 2299925 | + | 4 | No |
| BmSPH98 | 48582.99 | 9.0611 | nscaf3079 | 1559969 | 1567660 | + | 6 | No |
| BmSPH99 | 72835.36 | 6.5958 | nscaf2912 | 1250626 | 1268273 | - | 7 | No |
| BmSPH100 | 67483.13 | 8.706 | nscaf2838 | 2097976 | 2117915 | + | 5 | No |
| BmSP101 | 31078.88 | 7.6172 | nscaf2930 | 167068 | 170931 | + | 3 | No |
| BmSP102 | 109435.67 | 8.3757 | nscaf2964 | 1005068 | 1013261 | + | 2 | No |
| BmSPH103 | 32185.25 | 8.1977 | nscaf3058 | 372666 | 378085 | + | 16 | No |
| BmSPH104 | 33388.74 | 4.9636 | nscaf2902 | 3829585 | 3835181 | + | 18 | No |
| BmSP105 | 29292.34 | 4.7682 | nscaf1898 | 15640833 | 15645556 | - | 13 | Yes |
| BmSPH106 | 26162.88 | 7.3326 | nscaf2852 | 1757564 | 1762877 | + | 6 | No |
| BmSPH107 | 37569.54 | 7.5683 | nscaf3072 | 1893852 | 1900315 | - | 27 | No |
| BmSPH108 | 38965.85 | 9.4736 | nscaf2853 | 1836112 | 1849868 | - | 6 | No |
| BmSPH109 | 53237.15 | 7.8756 | nscaf3015 | 9461 | 12939 | + | 23 | Yes |
| BmSPH110 | 22641.32 | 9.6572 | nscaf2853 | 6760355 | 6763117 | + | 6 | Yes |
| BmSP111 | 57753.01 | 8.5184 | nscaf3027 | 5219844 | 5227462 | + | 23 | No |
| BmSPH112 | 18888.26 | 4.256 | nscaf2888 | 6476956 | 6479920 | - | 15 | No |
| BmSPH113 | 31683.68 | 8.4821 | nscaf2795 | 389364 | 394162 | - | 20 | No |
| BmSPH114 | 26518.16 | 5.1045 | nscaf2823 | 4197800 | 4204388 | - | 25 | No |
| BmSPH115 | 17832.02 | 4.06 | nscaf2828 | 3312323 | 3314479 | + | 8 | Yes |
| BmSP116 | 148347.97 | 6.7453 | nscaf2674 | 3915859 | 3926130 | + | 5 | No |
| BmSPH117 | 13432.06 | 8.3375 | nscaf2674 | 463799 | 468290 | + | 5 | Yes |
| BmSPH118 | 97067.44 | 9.3804 | nscaf3026 | 3146756 | 3159916 | + | 23 | Yes |
| BmSPH119 | 28855.87 | 7.8677 | nscaf2890 | 525073 | 531483 | - | 9 | Yes |
| BmSPH120 | 10896.32 | 7.5414 | scaffold846 | 14411 | 16589 | + | / | Yes |
| BmSPH121 | 37652.22 | 10.937 | nscaf2795 | 1705587 | 1710990 | + | 20 | Yes |
| BmSPH122 | 55198.22 | 8.8509 | nscaf2330 | 543585 | 554247 | - | 26 | No |
| BmSPH123 | 32506.52 | 7.9339 | nscaf2053 | 332355 | 333209 | + | 2 | Yes |
| BmSP124 | 43831.72 | 6.2825 | nscaf2986 | 3872648 | 3881630 | + | 7 | Yes |
| BmSPH125 | 42734.93 | 6.9262 | nscaf2912 | 1124441 | 1141084 | - | 7 | Yes |
| BmSP126 | 29721.33 | 10.6362 | scaffold769 | 10251 | 11882 | - | / | Yes |
| BmSPH127 | 42645.96 | 4.8078 | nscaf2823 | 4238746 | 4245308 | + | 25 | Yes |
| BmSPH128 | 203642.09 | 5.6917 | nscaf3154 | 27583 | 41256 | - | 9 | Yes |
| BmSPH129 | 45937.73 | 4.7331 | scaffold606 | 23519 | 38676 | + | 24 | Yes |
| BmSPH130 | 25413.36 | 9.1682 | nscaf2674 | 1720302 | 1723064 | - | 5 | Yes |
| BmSP131 | 44087.97 | 5.1346 | nscaf3044 | 472119 | 480531 | - | 21 | Yes |
| BmSP132 | 28520.68 | 4.952 | nscaf3098 | 586032 | 609689 | - | 28 | Yes |
| BmSPH133 | 47970.89 | 8.0915 | nscaf3097 | 2382599 | 2385848 | - | 28 | Yes |
| BmSP134 | 36437.02 | 6.8367 | nscaf2852 | 1714343 | 1720702 | - | 6 | Yes |
| BmSPH135 | 33677.48 | 8.1225 | nscaf2853 | 1893112 | 1935269 | - | 6 | Yes |
| BmSPH136 | 27624.48 | 9.1933 | nscaf1898 | 5237537 | 5239654 | - | 13 | No |
| BmSPH137 | 43345.41 | 5.6723 | nscaf2957 | 34451 | 45448 | + | / | Yes |
| BmSPH138 | 28154.76 | 5.9627 | nscaf2983 | 1075283 | 1077600 | - | 7 | Yes |
| BmSPH139 | 30825.94 | 5.1622 | nscaf2853 | 971877 | 985734 | - | 6 | Yes |
| BmSP140 | 26009.44 | 9.1609 | nscaf2674 | 1666921 | 1675086 | - | 5 | Yes |
| BmSP141 | 31923.63 | 8.0801 | nscaf2993 | 7842756 | 7843487 | + | 12 | Yes |
| BmSP142 | 34566.74 | 5.4605 | nscaf3058 | 7445256 | 7459846 | - | 16 | Yes |
| BmSPH143 | 34729.64 | 4.5096 | nscaf3058 | 7651604 | 7672371 | - | 16 | Yes |
