# Supplementary figures and images for: Genome-wide identification and expression analysis of serine proteases and homologs in the silkworm Bombyx mori
Source: BMC Genomics. 2010 Jun 24;11:405. doi: 10.1186/1471-2164-11-405 (PMC2996933; doi:10.1186/1471-2164-11-405)

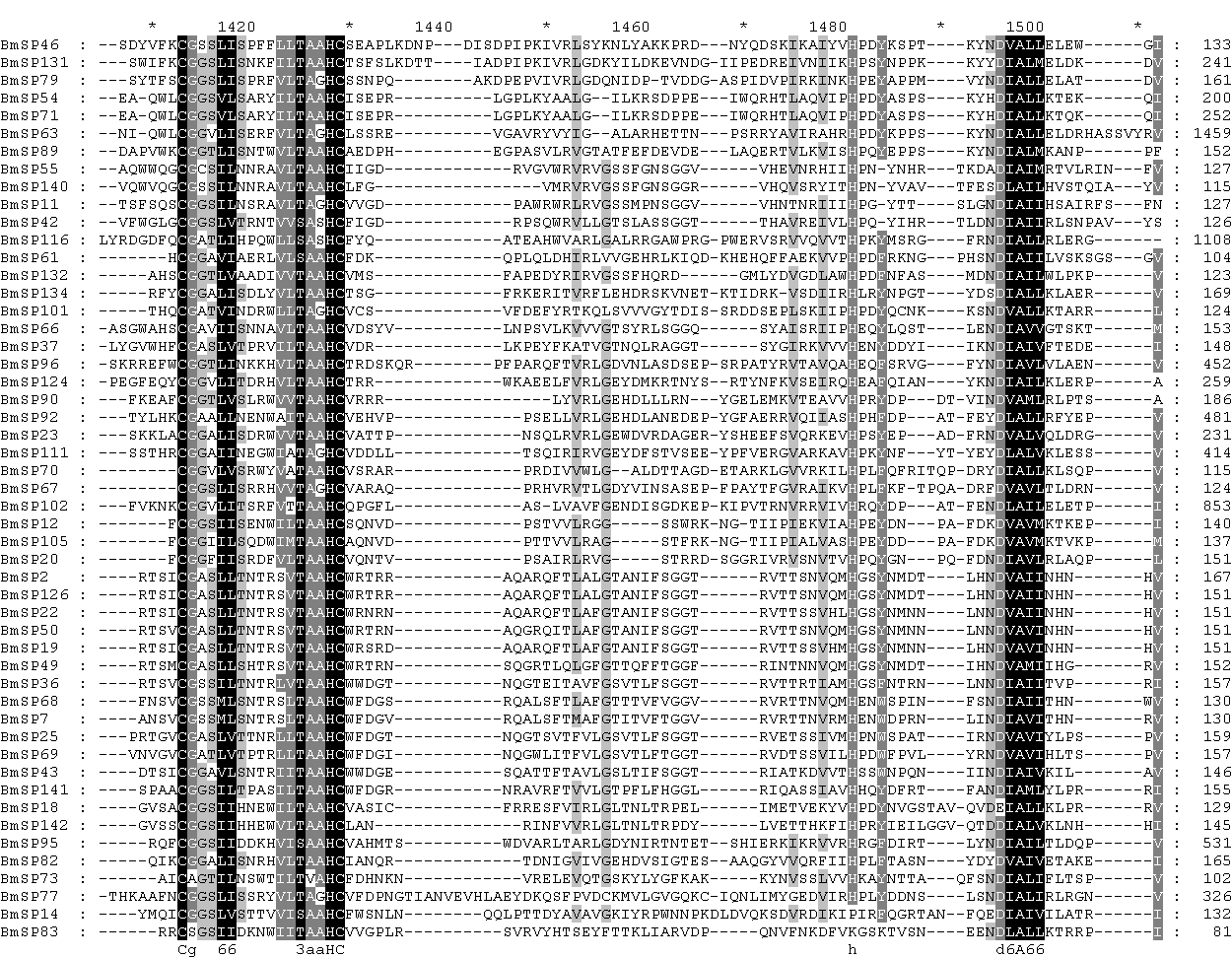

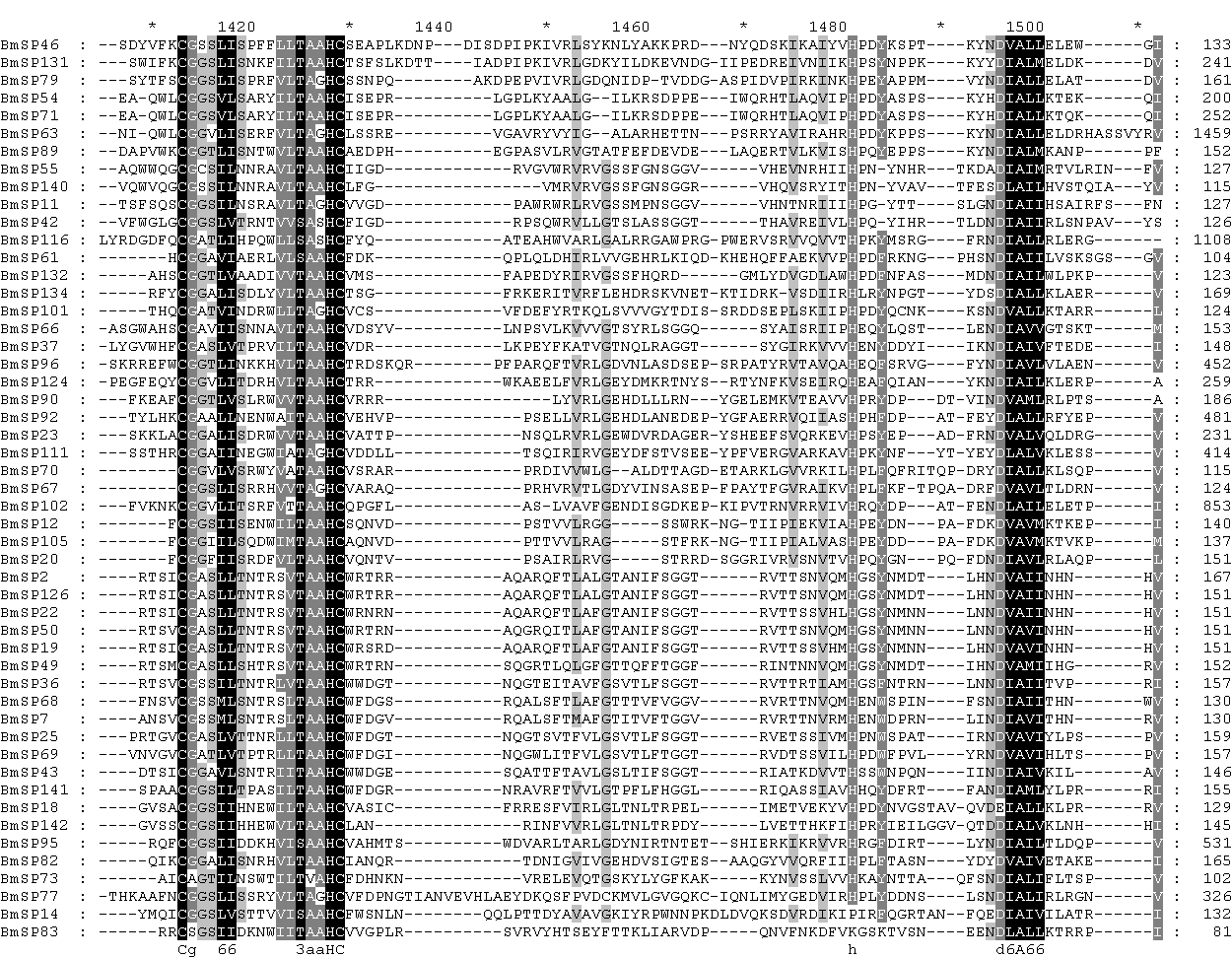

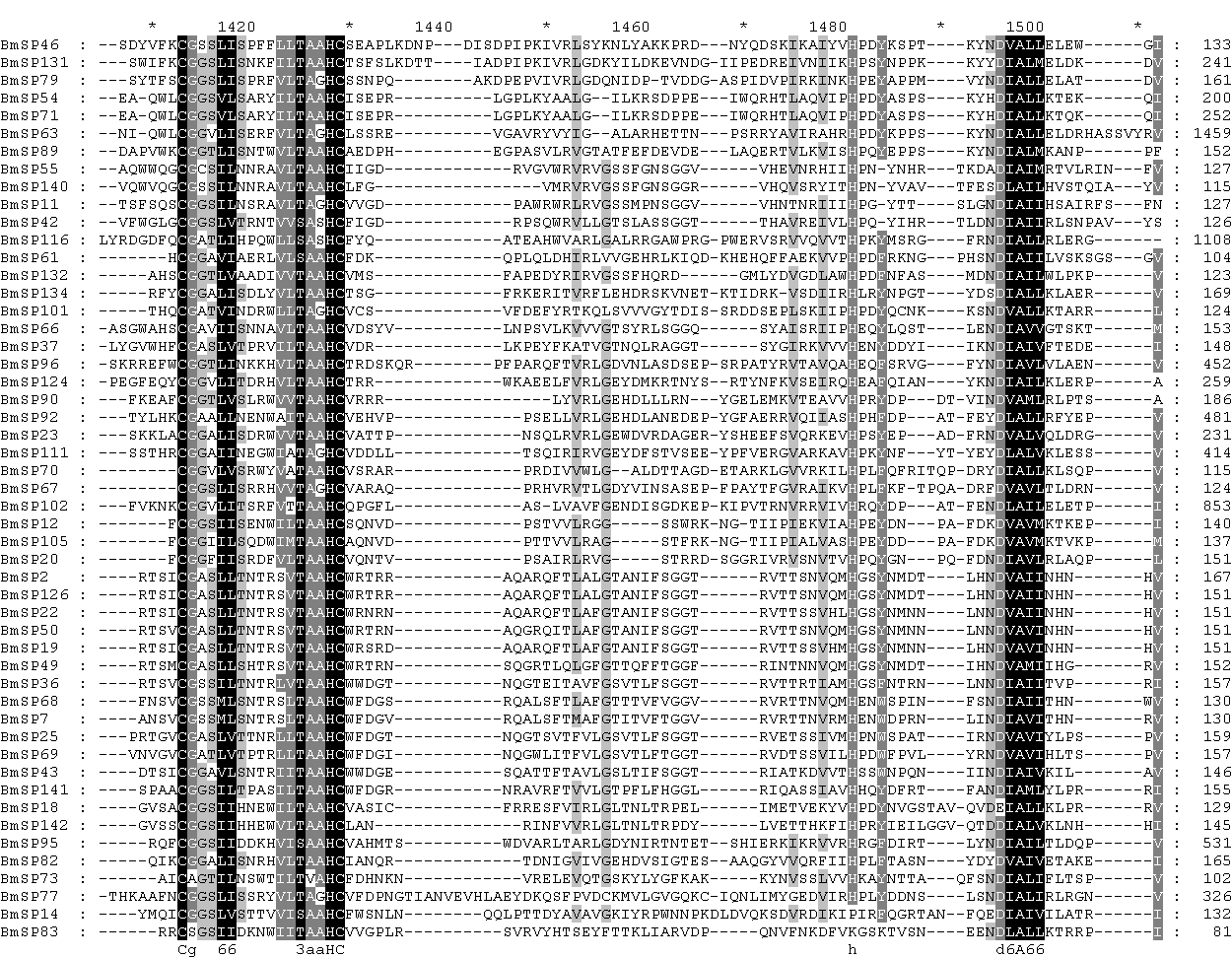

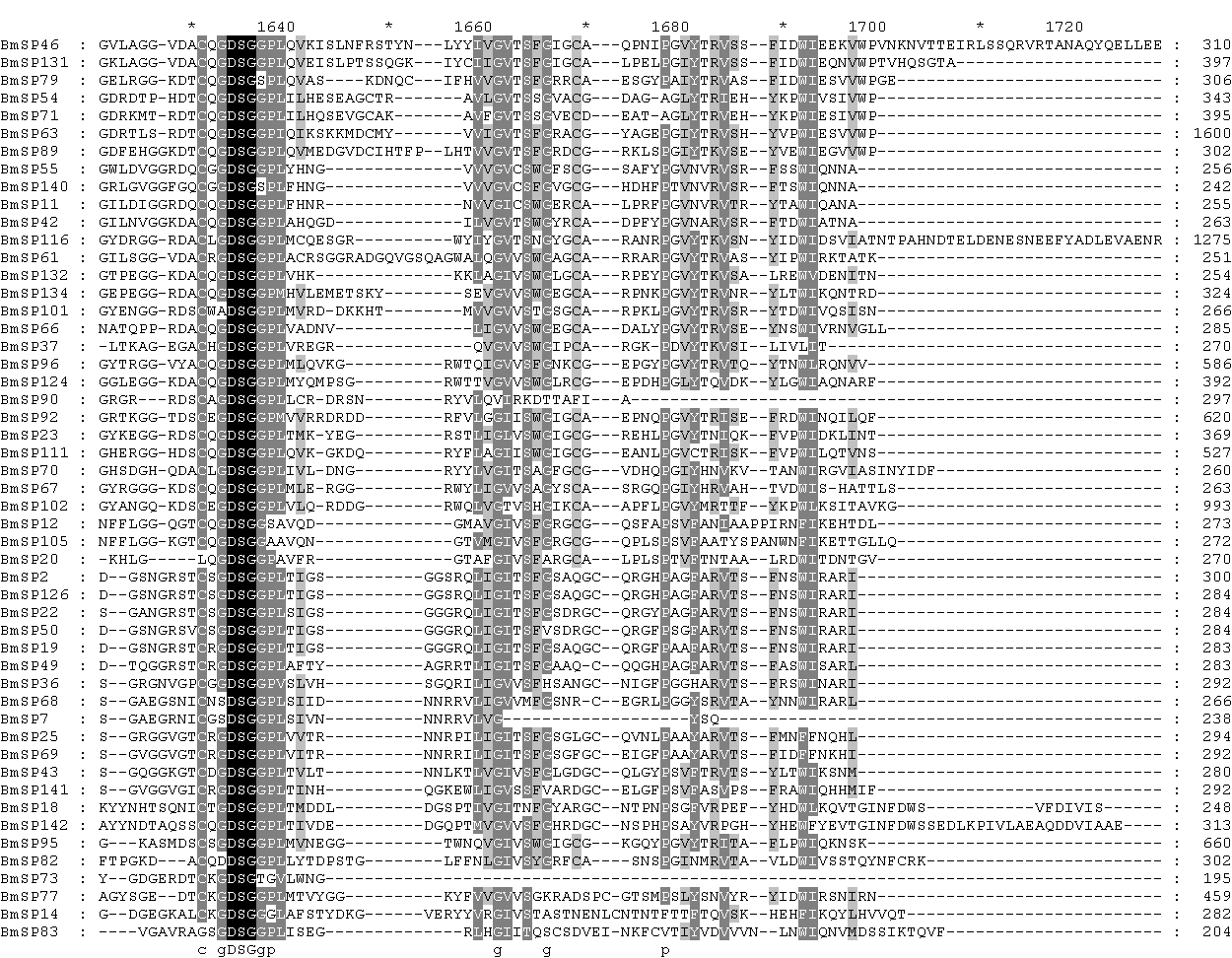


His

Asp

Ser

Supplement: Additional file 2 — The serine protease genes that contain intact active site . The active site of Ser, His, and Asp play important role in the catalytic processes for SP genes. By analyzing the gene sequences, we found that 51 serine protease genes have the three active site residues intact (SP), and the rest 92 serine protease genes had mutations in the catalytic residues (SPH), so they may have lost catalytic function. [file 1471-2164-11-405-S2.DOC]
