# Supplementary material for: Genome-wide identification and expression analysis of serine proteases and homologs in the silkworm Bombyx mori
Source: BMC Genomics. 2010 Jun 24;11:405. doi: 10.1186/1471-2164-11-405 (PMC2996933; doi:10.1186/1471-2164-11-405)
Supplement: Additional file 3 — The SPs and SPHs in the silkworm that have been reported . The GenBank accession numbers and description of the reported SPs and SPHs in the silkworm are listed. [file 1471-2164-11-405-S3.DOC]

| Name | GenBank Accession No. | Description |
| --- | --- | --- |
| BmSP124 | NP_001036915 | Serine protease [*Bombyx mori*] |
| BmSPH125 | NP_001036891 | Serine protease [*Bombyx mori*] |
| BmSP126 | NP_001036826 | Serine protease [*Bombyx mori*] |
| BmSPH127 | AAL31707 | Prophenoloxidase activating factor 3 |
| BmSPH128 | AAL62027 | Ovarian serine protease [*Bombyx mori*] |
| BmSPH129 | NP_001037053 | Serine protease homolog 1[*Bombyx mori*] |
| BmSPH130 | AAB26023 | Alkaliphilic serine protease P-IIc [*Bombyx mori* |
| BmSP131 | NP_001040537 | Serine protease 7 [*Bombyx mori]* |
| BmSP132 | BAA03758 | Vitellin-degrading protease precursor |
| BmSPH133 | NP_001036832 | Prophenoloxidase activating enzyme [*Bombyx mori*] |
| BmSP134 | BAG68694 | 37-kDa protease [*Bombyx mori*] |
| BmSPH135 | NP_001037368 | Serine protease-like protein[*Bombyx mori*] |
| BmSPH136 | ABR14241 | Cocoonase [*Bombyx mori*] |
| BmSPH137 | ABF51436 | Serine protease-like protein [*Bombyx mor*i] |
| BmSPH138 | ABF51402 | Trypsin-like protease [*Bombyx mori*] |
| BmSPH139 | ABF51356 | Scolexin [*Bombyx mori*] |
| BmSP140 | ABF51234 | Trypsin-like protease [*Bombyx mori*] |
| BmSP141 | ABD36200 | Chymotrypsinogen [*Bombyx mori*] |
| BmSP142 | NP_001037037 | 35kDa protease [*Bombyx mori*] |
| BmSPH143 | BAA77401 | 30kP protease A （43k peptide） |
