# Supplementary material for: Genome-wide identification and expression analysis of serine proteases and homologs in the silkworm Bombyx mori
Source: BMC Genomics. 2010 Jun 24;11:405. doi: 10.1186/1471-2164-11-405 (PMC2996933; doi:10.1186/1471-2164-11-405)
Supplement: Additional file 4 — The clip domain in the silkworm, Bombyx mori. The conserved six cysteine residues for each CLIP are marked by color. We identified 7 clip-SPs and 11 clip-SPHs in the silkworm, which are consisted of a chymotrypsin-like SP domain, and one or more clip domain(s) at the N-terminus. [file 1471-2164-11-405-S4.DOC]

>BmSP23/BmCLIP10

CKHRGARYPCGLSISCVLGGGKPLDLCSGGMIWA**CC**

>BmSPH34/BmCLIP12

CVRGGYNGTCVLSKRCETLILDYRDRSFPPVCGLRGKEPIVCC

>BmSP71/BmCLIP7

CVRDGYNGTCVLSKRCETLILDYRDRSFPPVCGLRGKEPIVCC

>BmSPH78/BmCLIP8

CTTPLGEASECVSLYDCSQLLSAFEQRPLQSKVVSFLRQSQCGFQGYVPRVCC

>BmSPH91/BmCLIP15

**C**V**C**VNYDQ**C**PSQEIIGRRDDLYLPIDPRNKGSEIVALTEEQLDNITNIEITQMTNTENSTN

DDVKKISKRDVKEDKPQEQAKEIEPRLFGSAGFGDNGVNTNKKVQPTFGVSFGLPQPSHGYPINPYGHSPIHNPYGPAINSGGINLGLLSVNPLLAIEVTKNDYGEKIVKPLVNLHITPNEHVVNRIGDLFHEKKHYLLNKHEHYHHHQPYHNSYGYYHHSHPPLVPTNHGPPIYSPHYTHYQNKYPGYFKSRPQLYESTASNEDDDYYNDDTDEHYNNEYDIHNPVHNNYGFERSLNVSTNDNGQKYANRYAYSRSLTVPQKPNGANPTGQTFRFPENRKKREINQSENFDQSSQRIQERQGYANRPLVQQ**C**RPNQV**CC**

>BmSP95-1

CVSTMGKKGTCKSFRDCYPLFKVADLSGWDGWVMGHYDTCSYISADNMEVFGVCC

>BmSP95-2

CVSTMGKKGTCKSFRDCYPLFKVADLSGWDGWVMGHYDTCSYISADNMEVFGVCC

>BmSP96/BmCLIP6

CTTPEGEEGICEDLSNCPQLLLNLINLRESLCFKDLFVPGVCC

>BmSPH99-1/BmCLIP14-1

CPGICKNAFAAYFCNKVLEEDDDACPDSSMKCC

>BmSPH99-2/BmCLIP14-2

CTGICVDTHLAQYCDAYFTAQDMCVKGKLCC

>BmSPH99-3/BmCLIP14-3

CKGECTGGLAALFCDRVDEDADCPNLETCC

>BmSPH99-4/BmCLIP14-4

CPGYCFLNLMAAFCPRPSVILTNTD CKNIGSLCC

>BmSPH99-5/BmCLIP14-5

CPGSCIVALLSFTCFRNAELTNVFKCKKTGTQCC

>BmSP102/BmCLIP5

CKASRVAKYNRGRTICMFNHECGQRGGEVVGACMDGFLFGACC

>BmSP111/BmCLIP9

CYQLLSGKSCKAGSAIGKCMFVQECIRQGGTHTGVCVDGFIFASCC

>BmSPH118

CAAALKCTPIEFCTAEGVISNTSVILSREQDAYRVPLTDCKDLGSGRIGKCC

>BmSP124/BmCLIP3

CTLPNGKAGRCRQLRHCIQEDFKKDYLVFMDYVCVIERSSIGVCC

>BmSPH125/BmCLIP4

CQTVENEVGSCLNLLQCTPYLKIVKEFKTNPAAAVLLRKAHCGFEGSNPKVCC

>BmSPH127/BmCLIP2

CNGGANCIPLEECTDLFQQLKQGNSPQLTRLLRGLHCGFEDLNSPKICC

>BmSPH129/BmCLIP11

CQTSDGQEGECVNYYLCNAANNTIITDGTNVIDIRVGSGPCSSYIDVCC

>BmSP133H-1/BmCLIP1-1

CRTPNGLNGNCVSVYECQALLAILNNQRRTQQDEKFLRDSQCGTKNSVPAVCC

>BmSP133H-2/BmCLIP1-2

CNAADGQQGNCVNINSCPYVLQLLKNPNEANLNYVRGSVCQGSEQQSICC

>BmSPH135

**C**GGANQENRIVGGMPASSNRYPWMARIVYDGQFH**C**GASVLTKEYVLTAAH**C**VKKLKRSKIRVILGDHDQTITSESAAIMRAVSAIVRHRSFDTESYNNDIALLKLRKPVNFSKIIKPV**C**LPPPKLRCC

>BmSPH137

CKCVPYYLCNKNNEGVDVNNASVTGWGVLDVRFGEEDCQESVEICC
