# Supplementary material for: Genome-wide identification and expression analysis of serine proteases and homologs in the silkworm Bombyx mori
Source: BMC Genomics. 2010 Jun 24;11:405. doi: 10.1186/1471-2164-11-405 (PMC2996933; doi:10.1186/1471-2164-11-405)
Supplement: Additional file 6 — The up-regulated SP and SPHs after silkworm infected by microorganisms . Genes whose expression were up-regulated than two fold on at least one occasion over the four time points in any of the four microorganism induction experiments were listed. The best NCBI BLAST Results of the up-regulated SP and SPHs were also shown in the table. [file 1471-2164-11-405-S6.DOC]

| Name | Best NCBI BLAST Results |
| --- | --- |
| BmSP42 | gb|ABU98619.1| protease [Helicoverpa armigera] Length=263 Score = 381 bits (979), Expect = 3e-104, Identities = 179/263 (68%), Positives = 225/263 (85%), Gaps = 0/263 (0%) |
| BmSP73 | ref|XP_001848040.1| urokinase-type plasminogen activator [Culex quinquefasciatus] Score = 98.2 bits (243), Expect = 3e-19,Identities = 63/198 (31%), Positives = 95/198 (47%), Gaps = 8/198 (4%) |
| BmSP36 | gb|AAX39408.1| serine protease [Bombyx mandarina] Length=284 Score = 282 bits (722), Expect = 2e-74, Identities = 148/267 (55%), Positives = 179/267 (67%), Gaps = 4/267 (1%) |
| BmSP43 | gb|AAC36150.1| chymotrypsinogen-like protein [Plodia interpunctella] Length=282 Score = 317 bits (811), Expect = 1e-84, Identities = 150/263 (57%), Positives = 195/263 (74%), Gaps = 4/263 (1%) |
| BmSP66 | gb|AAB66878.1| trypsin [Anopheles stephensi] Length=274 Score = 197 bits (501), Expect = 1e-48, Identities = 106/236 (44%), Positives = 145/236 (61%), Gaps = 10/236 (4%) |
| BmSPH107 | ref|XP_555167.1| AGAP008295-PA [Anopheles gambiae str. PEST] Score = 184 bits (467), Expect = 9e-45, Identities = 98/237 (41%), Positives = 142/237 (59%), Gaps = 16/237 (6%) |
| BmSP25 | gb|ABR88238.1| chymotrypsin-like protease C8 [Heliothis virescens] Length=293 Score = 352 bits (902), Expect = 3e-95, dentities = 176/297 (59%), Positives = 220/297 (74%), Gaps = 8/297 (2%) |
| BmSPH10 | emb|CAL92020.1| chymotrypsinogen-like protein 1 [Manduca sexta] Length=281 Score = 385 bits (988), Expect = 8e-105, Identities = 185/263 (70%), Positives = 213/263 (80%), Gaps = 2/263 (0%) |
| BmSPH32 | gb|ABR88231.1| chymotrypsin-like protease C1 [Heliothis virescens] Length=279 Score = 325 bits (833), Expect = 2e-87, Identities = 155/265 (58%), Positives = 192/265 (72%), Gaps = 26/265 (9%) |
| BmSP46 | gb|AAV91007.1| hemolymph proteinase 9 [Manduca sexta] Length=393 Score = 330 bits (845), Expect = 4e-88,Identities = 161/272 (59%), Positives = 195/272 (71%), Gaps = 12/272 (4%) |
| BmSP141 | ref|NP_001040178.1| chymotrypsinogen [Bombyx mori] Length=292 Score = 504 bits (1299), Expect = 2e-141, Identities = 243/243 (100%), Positives = 243/243 (100%), Gaps = 0/243 (0%) |
| BmSPH4 | gb|ABU98624.1| protease [Helicoverpa armigera] Length=260 Score = 261 bits (667), Expect = 3e-68,Identities = 129/198 (65%), Positives = 149/198 (75%), Gaps = 3/198 (1%) |
| BmSPH44 | gb|AAK81696.1| trypsin-like protein [Galleria mellonella] Length=255 Score = 172 bits (436), Expect = 1e-41, Identities = 87/195 (44%), Positives = 119/195 (61%), Gaps = 8/195 (4%) |
| BmSPH75 | gb|AAV91007.1| hemolymph proteinase 9 [Manduca sexta] Length=393 Score = 208 bits (530), Expect = 3e-52, Identities = 101/178 (56%), Positives = 129/178 (72%), Gaps = 1/178 (0%) |
| BmSP131 | ref|NP_001040537.1| serine protease 7 [Bombyx mori] Length=397 Score = 823 bits (2126), Expect = 0.0, Identities = 396/397 (99%), Positives = 397/397 (100%), Gaps = 0/397 (0%) |
| BmSP95 | ref|XP_972363.2| PREDICTED: similar to trypsin-like serine protease [Tribolium castaneum] Length=539 Score = 438 bits (1126), Expect = 1e-120, Method: Compositional matrix adjust.Identities = 228/431 (52%), Positives = 272/431 (63%), Gaps = 59/431 (13%) |
| BmSPH87 | ref|XP_001998666.1| GI24097 [Drosophila mojavensis] Length=262 Score = 83.6 bits (205), Expect = 5e-15, Identities = 38/61 (62%), Positives = 48/61 (78%), Gaps = 3/61 (4%) |
| BmSPH125 | E-value:0.0 Annotation:ref|NP_001036891.1| serine protease [Bombyx mori] dbj|BAB91156.1| serine protease [Bombyx mori]" |
