# Supplementary material for: Genome-wide identification and expression analysis of serine proteases and homologs in the silkworm Bombyx mori
Source: BMC Genomics. 2010 Jun 24;11:405. doi: 10.1186/1471-2164-11-405 (PMC2996933; doi:10.1186/1471-2164-11-405)
Supplement: Additional file 7 — The induced expression analysis of silkworm SP and SPH genes by quantitative real-time RT-PCR . We chose the time points of infecting 6 h and 24 h to do the expression analysis. The expression of SP or SPH in the control sample was set to 1. The abbreviations are used, the E. coli infected sample (Ec), the B. bombyseptieus infected sample (Bs), the B. bassiana infected sample (Bb) and the B. mori nucleopolyhedrovirus infected sample (NPV). Each expressive assay was replicated by three times. The Student's t-test was used to evaluate statistical signicance (P < 0. 01). [file 1471-2164-11-405-S7.DOC]

**Figure continued**
