# Supplementary material for: Genome-wide identification and expression analysis of serine proteases and homologs in the silkworm Bombyx mori
Source: BMC Genomics. 2010 Jun 24;11:405. doi: 10.1186/1471-2164-11-405 (PMC2996933; doi:10.1186/1471-2164-11-405)
Supplement: Additional file 8 — Primers used in semi-quantitative RT-PCR study . Primer sequences, melting temperature, and amplicon size were listed. [file 1471-2164-11-405-S8.DOC]

| Gene | (5'-3') | Primer | Size(bp) | Melting temperature(℃) |
| --- | --- | --- | --- | --- |
| BmSP95 | Forward | ATCACGAAACCACCACAAG | 558 | 56 |
|  | Reverse | CGGTAAACAAATGGGACGA |  |  |
| BmSPH104 | Forward | GTAGTGGGCGAATGGGATA | 437 | 58 |
|  | Reverse | CGTTTGATGAGTAGTGGTGC |  |  |
| BmSPH128 | Forward | AGAAGGCACCGTATTCCC | 532 | 56 |
|  | Reverse | CAGTCGCATTAACACCATC |  |  |
| BmSP25 | Forward | TTTCCTTATCAGGGTGGTC | 585 | 56 |
|  | Reverse | CAAGATAGGACGGTTGTTTC |  |  |
| BmSP36 | Forward | GCTCACTGCTGGTGGGATG | 529 | 58 |
|  | Reverse | CCGGGAAGCCAATGTTGC |  |  |
| BmSP43 | Forward | AGGATTGTCGGAGGAAGT | 436 | 54 |
|  | Reverse | TTGACGAAGGGAAAGATGC |  |  |
| BmSPH47 | Forward | CCCCAAGCCTGAAGATGA | 593 | 56 |
|  | Reverse | TGTGAGGTTCGACAGGAGG |  |  |
| BmSPH85 | Forward | CCTCCAAGATGTCCGTTTC | 475 | 58 |
|  | Reverse | TTCTTCAGGAGCCAGGGTA |  |  |
| BmSP141 | Forward | GCGCCAATCAATTACCAT | 438 | 51 |
|  | Reverse | AACATTTCTCGCCATCGT |  |  |
| BmSP142 | Forward | TTGGGCTTGACGAACCTG | 424 | 58 |
|  | Reverse | TCAAAGGACCACCACTGTCT |  |  |
