# Supplementary material for: Genome-wide identification and expression analysis of serine proteases and homologs in the silkworm Bombyx mori
Source: BMC Genomics. 2010 Jun 24;11:405. doi: 10.1186/1471-2164-11-405 (PMC2996933; doi:10.1186/1471-2164-11-405)
Supplement: Additional file 9 — Primers used in qRT-PCR study . Primer sequences, melting temperature and amplicon size were listed. [file 1471-2164-11-405-S9.DOC]

| Gene | (5'-3') | Primer | Size(bp) | Melting temperature(℃) |
| --- | --- | --- | --- | --- |
| BmSP36 | Forward | GGCTGAAGGTATTCCCGCT | 169 | 58 |
|  | Reverse | AGAGGACCCGCACACTGAG |  |  |
| BmSP73 | Forward | ATTTAGGTGCTATTTGTGCTG | 123 | 52 |
|  | Reverse | ATAAGTATTTTGAACCCGTCT |  |  |
| BmSP43 | Forward | CATTTACCTTTGCGATTGG | 119 | 54 |
|  | Reverse | AACCTGACTTCCTCCGACA |  |  |
| BmSP66 | Forward | ATCATACGAAGGCACCACA | 117 | 53 |
|  | Reverse | CTACTAAAGGACCACCCGA |  |  |
| BmSPH107 | Forward | TGGAAAGAGTCAATCGGCT | 105 | 55 |
|  | Reverse | CTTGGTCTCGGGTTGGGCT |  |  |
| BmSP25 | Forward | GCTTACGGCAGTGCTTTTA | 103 | 55 |
|  | Reverse | CTTCAGCCTTGCGAATCCT |  |  |
| BmSPH10 | Forward | TCCCCAAGCCTGAAGATGA | 157 | 58 |
|  | Reverse | ACTGAGCCTCCGCAGACAA |  |  |
| BmSPH32 | Forward | TAAAGACGCATTCCCCAAG | 231 | 55 |
|  | Reverse | GCACCTGAAGAAAAGACCG |  |  |
| BmSP42 | Forward | TTGCGGTGCCAAGAAGTGT | 165 | 59 |
|  | Reverse | AATGGGAAGCGGAGACGAC |  |  |
| BmSP46 | Forward | GGAGCGAATAGACACTGAAGAT | 174 | 56 |
|  | Reverse | AAAAATGGACTGATTAGGGAGC | |  |
| BmSP141 | Forward | TTTGGCAGGTCTACTGATTGA | 221 | 52 |
|  | Reverse | TGAAGTCGTATTGATGATGAA |  |  |
| BmSPH4 | Forward | ACATTCCGTAACGCCAGT | 72 | 52 |
|  | Reverse | GAGCAAGCCATAGGTAGG |  |  |
| BmSPH44 | Forward | ATCATAAGCCGCATCACG | 108 | 55 |
|  | Reverse | GACGAGACCAGCCACGAA |  |  |
| BmSP131 | Forward | AACCACCGAAGAACCTGAA | 197 | 55 |
|  | Reverse | GTCCCCCAATAGCAAATGA |  |  |
| BmSPH75 | Forward | ATTTGCGATGGTTACAGTTTG | 257 | 56 |
|  | Reverse | AGGTTCAGTCCAGAGACAGGC |  |  |
| BmSPH87 | Forward | CATAGACCCCGCATCCAT | 80 | 55 |
|  | Reverse | GACCTCCGCTGTCACCTT |  |  |
| BmSPH125 | Forward | CGATTTCCCTTGGATGGT | 240 | 54 |
|  | Reverse | TCGTATTGCTCGTGCTTG |  |  |
