# Supplementary material for: Genome-wide identification and expression analysis of serine proteases and homologs in the silkworm Bombyx mori
Source: BMC Genomics. 2010 Jun 24;11:405. doi: 10.1186/1471-2164-11-405 (PMC2996933; doi:10.1186/1471-2164-11-405)
Supplement: Supplementary file 9 [file 1471-2164-11-405-S5.DOC]

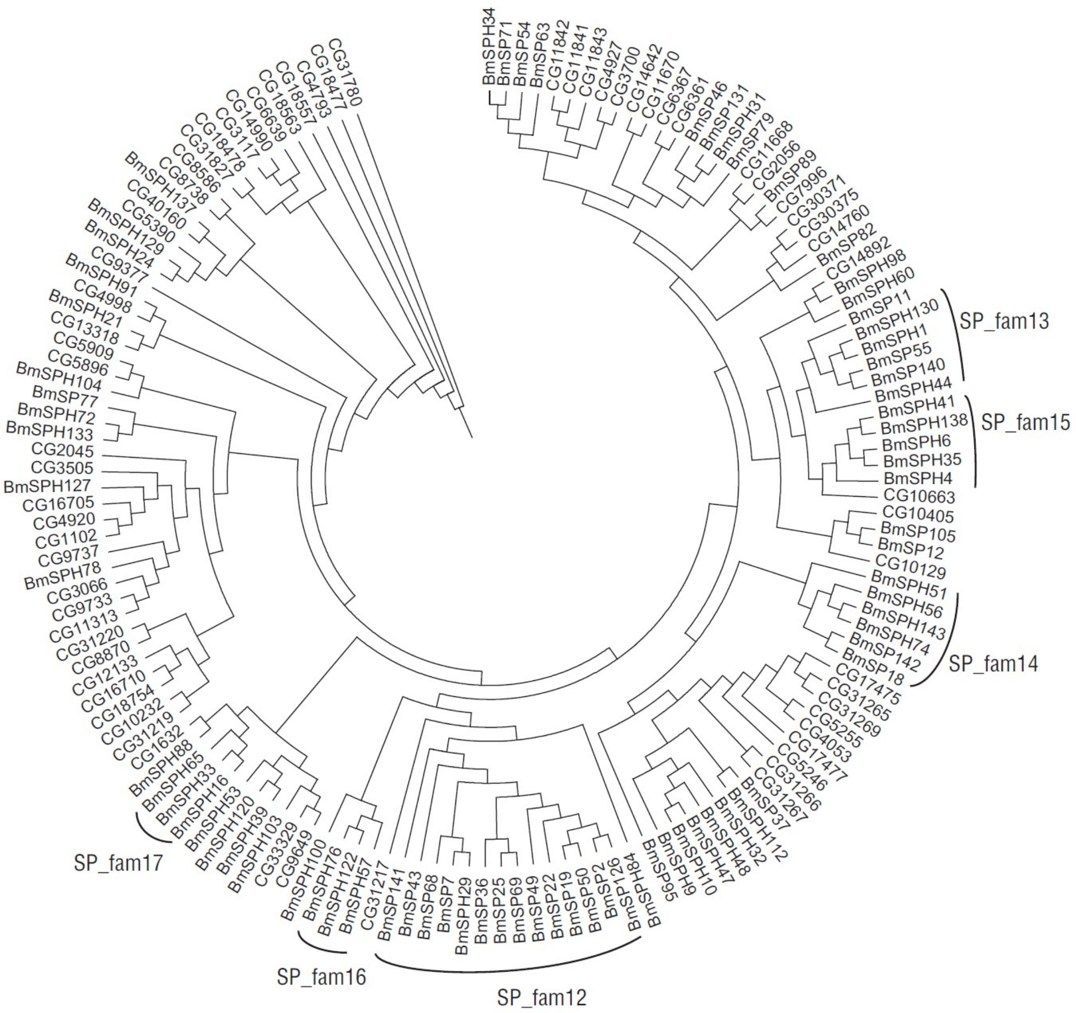
BmSPH34
BmSP71
BmSP54
BmSP63
CG11842
CG11841
CG11843
CG4927
CG3700
CG14642
CG11670
CG6367
CG6361
BmSP46
BmSPH131
BmSP79
CG11668
CG2056
BmSP89
CG7996
CG30371
CG30375
CG14760
BmSP82
CG14892
BmSPH98
BmSPH60
BmSP11
BmSPH130
BmSPH1
BmSP55
BmSP140
BmSPH44
SP_fam13
BmSPH41
BmSPH138
BmSPH6
BmSPH35
BmSPH4
SP_fam15
CG10663
CG10405
BmSP105
BmSP12
CG10129
BmSPH51
BmSPH56
BmSPH143
BmSPH74
BmSPH142
BmSP18
SP_fam14
CG17475
CG31265
CG31269
CG5255
CG4053
CG5246
CG17477
CG31266
CG31267
BmSP37
BmSPH112
BmSPH32
BmSPH48
BmSPH47
BmSPH10
BmSPH9
BmSP95
BmSPH84
BmSP126
BmSP2
BmSP50
BmSP19
BmSP22
BmSP49
BmSP69
BmSP25
BmSP36
BmSPH29
BmSP7
BmSP68
BmSP43
BmSP141
SP_fam12
CG31217
BmSPH57
BmSPH122
BmSPH76
SP_fam16
BmSPH100
CG9649
CG33329
BmSPH103
BmSPH39
BmSPH120
BmSPH53
BmSPH16
BmSPH33
BmSPH65
BmSPH88
SP_fam17
CG1632
CG31219
CG10232
CG18754
CG16710
CG12133
CG8870
CG31220
CG11313
CG9733
CG3066
BmSPH78
CG9737
CG1102
CG4920
CG16705
BmSPH127
CG3505
CG2045
BmSPH133
BmSPH72
BmSP77
BmSPH104
CG5896
CG5909
CG13318
BmSPH21
CG4998
BmSPH91
CG9377
BmSPH24
BmSPH129
CG5390
CG40160
BmSPH137
CG8738
CG8586
CG31827
CG18478
CG3117
CG14990
CG6639
CG18563
CG18557
CG4793
CG18477
CG31780
